# Transcriptional Analysis of T Cells Resident in Human Skin

**DOI:** 10.1371/journal.pone.0148351

**Published:** 2016-01-29

**Authors:** Jane Li, Moshe Olshansky, Francis R. Carbone, Joel Z. Ma

**Affiliations:** 1 Department of Microbiology and Immunology, The University of Melbourne at The Peter Doherty Institute for Infection and Immunity, Parkville, Victoria, Australia; 2 Department of Medicine (St Vincent’s Hospital), The University of Melbourne, Fitzroy, Victoria, Australia; Leiden University Medical Center, NETHERLANDS

## Abstract

Human skin contains various populations of memory T cells in permanent residence and in transit. Arguably, the best characterized of the skin subsets are the CD8^+^ permanently resident memory T cells (T_RM_) expressing the integrin subunit, CD103. In order to investigate the remaining skin T cells, we isolated skin-tropic (CLA^+^) helper T cells, regulatory T cells, and CD8^+^ CD103^-^ T cells from skin and blood for RNA microarray analysis to compare the transcriptional profiles of these groups. We found that despite their common tropism, the T cells isolated from skin were transcriptionally distinct from blood-derived CLA^+^ T cells. A shared pool of genes contributed to the skin/blood discrepancy, with substantial overlap in differentially expressed genes between each T cell subset. Gene set enrichment analysis further showed that the differential gene profiles of each human skin T cell subset were significantly enriched for previously identified T_RM_ core signature genes. Our results support the hypothesis that human skin may contain additional T_RM_ or T_RM_-like populations.

## Introduction

Human skin at steady state contains a vast number of memory T cells [1]. Traditionally, memory T cells have been divided into two populations: central memory T cells (T_CM_) that circulate mainly between the lymphoid tissues and effector memory T cells (T_EM_) that migrate to extralymphoid peripheral tissues [2]. T_CM_ and T_EM_ are distinguished by the expression of CCR7 and CD62L, or lack thereof (T_CM_−CCR7^+^CD62L^+^, T_EM_−CCR7^-^CD62L^-^), and both may be found in normal human skin [1]. Recently, a subset of CD8^+^ T cells has been discovered that resides permanently in peripheral tissues post-infection, without returning to the circulation [3–5]. These T cells provide accelerated long-lived site-specific immunity and have been termed resident memory T cells (T_RM_) [3,5,6]. T_RM_ are generally defined by surface expression of CD103 (α_E_ integrin) and CD69 but lack of CCR7 and CD62L, and have been described in both mice and humans in many non-lymphoid tissues such as gut, brain, lung, skin and genital mucosa [3,7–11]. Since their discovery, CD8^+^CD103^+^ T_RM_ have been studied extensively. Microarray analyses in mouse models have identified the transcriptomes of these CD8^+^CD103^+^ T_RM_ in several tissues, including skin [7,12], demonstrating that these T_RM_ are a separate subset distinct from T_CM_ and T_EM_.

Apart from CD8^+^CD103^+^ T_RM_, skin contains other T_RM_, as well as a heterogeneous population of recirculating memory T cells (T_RCM_) comprising T_EM_, T_CM_, and other subsets yet to be described in detail [13,14]. T_RCM_ presumably recirculate between blood and skin through the expression of skin addressins such as cutaneous lymphocyte antigen (CLA), CCR4 and CCR10 [15,16]. Studies in murine skin have found CD4^+^CCR7^+^ T_RCM_ with effector functions more akin to T_CM_ than T_EM_ [14], and CD4^+^ regulatory T cells (T_reg_) which reversibly traffic between skin and blood [17]. Interestingly, these experiments also identified a subset of CD4^+^CD103^+^CCR7^-^ T cells that did not reenter the circulation, suggesting that the skin may also harbour CD4^+^ T_RM_ [14]. A comparable complexity appears to exist in human skin. In a study of patients with cutaneous T cell lymphoma treated with the monoclonal antibody alemtuzumab, which depletes circulating T cells but spares T_RM_, both CD8^+^ and CD4^+^ T cells, including T_reg_, persisted in the skin [13]. Thus, the present literature indicates that skin contains multiple T cell subsets, some of which have yet to be fully defined.

We sought to further characterize human skin T_RM_ and T_RCM_ by undertaking a gene expression microarray analysis of skin-tropic memory T cells in blood compared to non-CD8^+^CD103^+^ T cells in the skin. We reasoned that T cells in skin would comprise both T_RM_ and T_RCM_, while the skin-tropic memory T cells in blood would comprise only T_RCM_. Our aim was to identify a gene expression “signature” that distinguished cutaneous CD8^+^ T cells, CD4^+^ T cells and T_reg_ from their blood equivalents. A secondary aim was to compare the transcriptional profile of these skin T cell groups with the currently known core signature of CD8^+^CD103^+^ T_RM_ in mouse models. We showed that skin-tropic T cells derived from skin and blood had distinct patterns of gene expression, with a shared pool of genes contributing to the skin/blood discrepancy. We also found that the human skin T cells were significantly enriched for established T_RM_ core signature genes compared to human blood T cells.

## Materials and Methods

### Tissue sample collection and pooled cell suspension preparation

The IMMGEN protocol (http://www.immgen.org) was consulted in the design of this microarray experiment. Peripheral blood mononuclear cells (PBMC) were obtained from 15 healthy donors (age range 17–72) and human skin samples were obtained as surgical discard from 15 healthy volunteers (age range 18–64). All donors were female to avoid gender-based disparities. The University of Melbourne human ethics committee approved this study and patients provided written informed consent. PBMC were acquired as cryopreserved samples from the Victorian Cancer Biobank or Australian Red Cross Blood Service. PBMC samples from donors aged 17 were from the Australian Red Cross Blood Service. Written consent was obtained directly from these donors and not from the next of kin as the minimum age for blood donation (parental consent not required) in Australia is 16 years.

Fresh skin samples were incubated in collagenase type 3 (3mg/ml; Worthington) with 5μg/ml DNAse in RPMI medium containing 2% (v/v) FCS for 1.5 hours at 37°C. Collagenase-digested tissue samples were then dissociated using the Becton Dickinson (BD) Medimachine into cell suspensions. To reduce the effect of inter-individual variability, pooled cell suspensions of PBMC or skin cells were obtained by combining samples from 3 donors. Cells were pooled such that the cumulative age of the donors was similar across all pools. The pooled cell suspensions were subjected to negative magnetic selection using the Dynabeads Untouched Human T cells Kit (Thermo Fisher Scientific) to deplete contaminating B cells, NK cells, monocytes, platelets, dendritic cells, granulocytes and erythrocytes.

### Fluorescence activated cell sorting and antibodies

**Fig 1:**
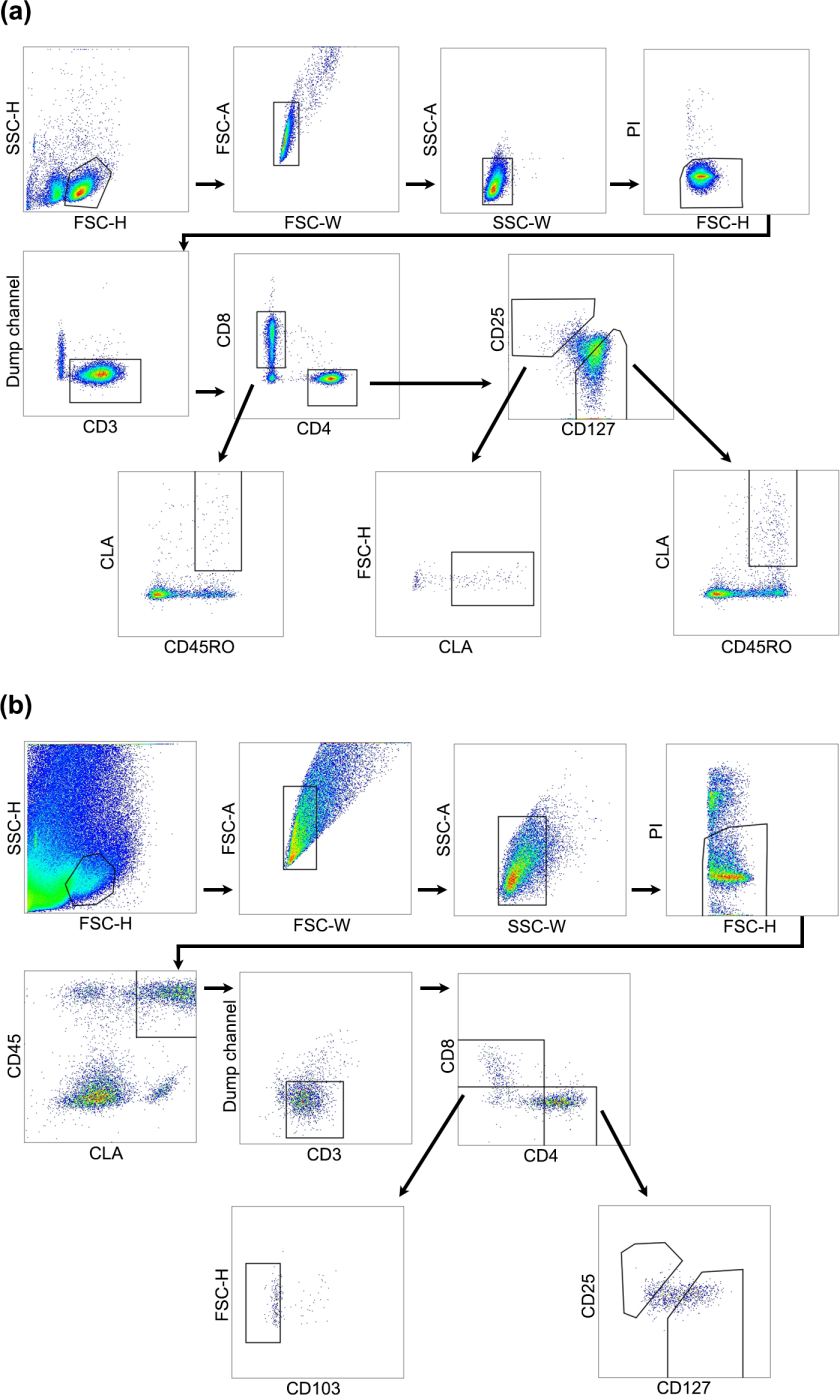
Multiple subsets of skin-tropic T cells are present in human skin and blood. Representative images are shown to demonstrate gating strategy for fluorescence activated cell sorting of T cells from blood and skin. In each case, gates were used to exclude debris, doublets and non-viable cells, and a dump channel was used to exclude irrelevant cell types. (a) For blood samples, T cells were identified based on CD3 expression, then divided into CD4^+^ and CD8^+^ populations. Skin-tropic CD8^+^ and CD4^+^ memory T cells were isolated based on their expression of memory marker CD45RO and skin addressin CLA. Skin-tropic regulatory T cells (T_reg_) were identified based on their CD25_hi_CD127_lo_ surface profile. (b) For skin samples, CD45 was used to eliminate cells of non-haematopoietic origin. CLA^+^ T cells were identified based on CD3 expression. CD8^+^CD103^-^ T cells were captured; CD4^+^ T cells (CD127_hi_) and T_reg_ (CD25_hi_CD127_lo_) were identified on the basis of CD25 and CD127 expression. FSC = forward scatter; PI = propidium iodide; SSC = side scatter.

Pooled cell suspensions were stained with a cocktail of fluorescence-conjugated antibodies for 30 minutes at 4°C. BD FACSAria III was used to collect live sorted cells at the Melbourne Cytometry ImmunoID Flow Cytometry facility (The University of Melbourne). The live cell populations collected are shown in S1 Table and representative gating is shown in Fig 1. Antibodies used from BD were: anti-CD8a (RPA-T8), anti-CD3 (UCHT1), anti-CLA (HECA-452), anti-CD25 (M-A251). Antibodies from eBioscience were: anti-CD4 (OKT4), anti-CD127 (eBioRDR5), anti-CD103 (BerACT8; used for skin samples only). Anti-CD45RO (UCHL1) antibody was obtained from Biolegend. To further insure against non-T cells contaminating the sort, a dump channel was created to exclude cells binding PerCP-Cyanine5.5-conjugated antibodies (all obtained from Biolegend) targeting CD138 (epithelial cells; used for skin samples only), CD235a (erythrocytes), CD14 (monocytes), CD19 (B cells), CD335 (NKp46; NK cells). Propidium iodide was used as a viability marker.

### RNA extraction, whole transcriptome amplification and microarray hybridization

After sorting, RNA was extracted using the Qiagen RNEasy Micro kit according to manufacturer’s instructions, then stored immediately at -80°C. RNA quality analysis, amplification, microarray hybridization and data acquisition were performed at the Ramaciotti Centre for Genomics at the University of New South Wales. In brief, RNA quality was assessed using a Bioanalyzer 2100 (Agilent). RNA was then used to generate complementary DNA (cDNA) through whole-transcriptome amplification using the Ovation PicoSL WTA V2 linear RNA amplification system (NuGEN). The cDNA was then labeled with the Encore Biotin module (NuGEN) and hybridized to HumanHT 12v4 BeadArrays (Illumina) prior to data acquisition.

### Microarray data analysis

Data was extracted from the raw intensity files from the Illumina HumanHT 12v4 BeadArrays using Bioconductor R limma package [18]. Bioconductor illuminaHumanv4.db package [19,20] (reannotation) was used to exclude bad probes and to accurately assign probes to transcripts. Due to high sample variability a standard Differential Expression analysis (like in limma package) did not produce satisfactory results, therefore RUVinv method (from ruv R package [21]) was used to generate log_2_transformed fold-change and corresponding p-values for pairwise comparisons [22,23]. A log_2_fold-change cut-off of ≥ 1.5 and a p-value cut-off of ≤ 0.05 after Benjamini-Hochberg false discovery rate multiple testing correction were used to identify significantly differentially expressed transcripts. Microarray data was submitted to the Gene Expression Omnibus (accession code GSE74158).

The PANTHER (protein analysis through evolutionary relationship) Classification System [24] version 10.0, 2015 (http://pantherdb.org) was used for gene ontology (GO) analysis. The PANTHER Statistical overrepresentation test with GO-Slim Biological Process annotation data was used; GO terms with p≤0.05 after Bonferroni correction were deemed significant. Pathway analysis was conducted with PathVisio version 3.2.1 software [25] using curated biological pathways from WikiPathways [26]. Gene sets for Gene Set Enrichment Analysis (GSEA) were obtained of genes upregulated and downregulated in murine skin, lung and gut T_RM_ [7] (for complete lists see S2 Table). GSEA was performed on log_2_transformed and quantile normalized data using GSEA v2.2.0 software from the Broad Institute [27]. Principal component analysis was performed on the normalized data and visualized using XLSTAT (Addinsoft SARL, US).

### Quantitative PCR

Quantitative (q)PCR was performed on the StepOne Plus system (Thermo Fisher Scientific) using 1–3 cDNA samples from each category of sorted cells (blood CD4^+^ = 3 samples; skin CD4^+^ = 1 sample; rest = 2 samples). Taqman Gene Expression Assays (Thermo Fisher Scientific) were obtained for *NR4A2* (Hs00428691_m1), *S1PR1* (Hs00173499_m1), *CCR7* (Hs01013469_m1), *CD8A* (Hs00233520_m1), *CD4* (Hs01058407_m1) and *CTLA4* (Hs03044418_m1), as well as *UBC* (Hs00824723_m1) and *B2M* (Hs00984230_m1), with the latter two used as housekeeping genes. Raw gene expression data was normalized to the geometric mean of housekeeping gene expression using the 2^-ΔCт^ comparative C_T_ method [28].

## Results

### Memory T cells in the skin share a common gene expression signature

**Fig 2:**
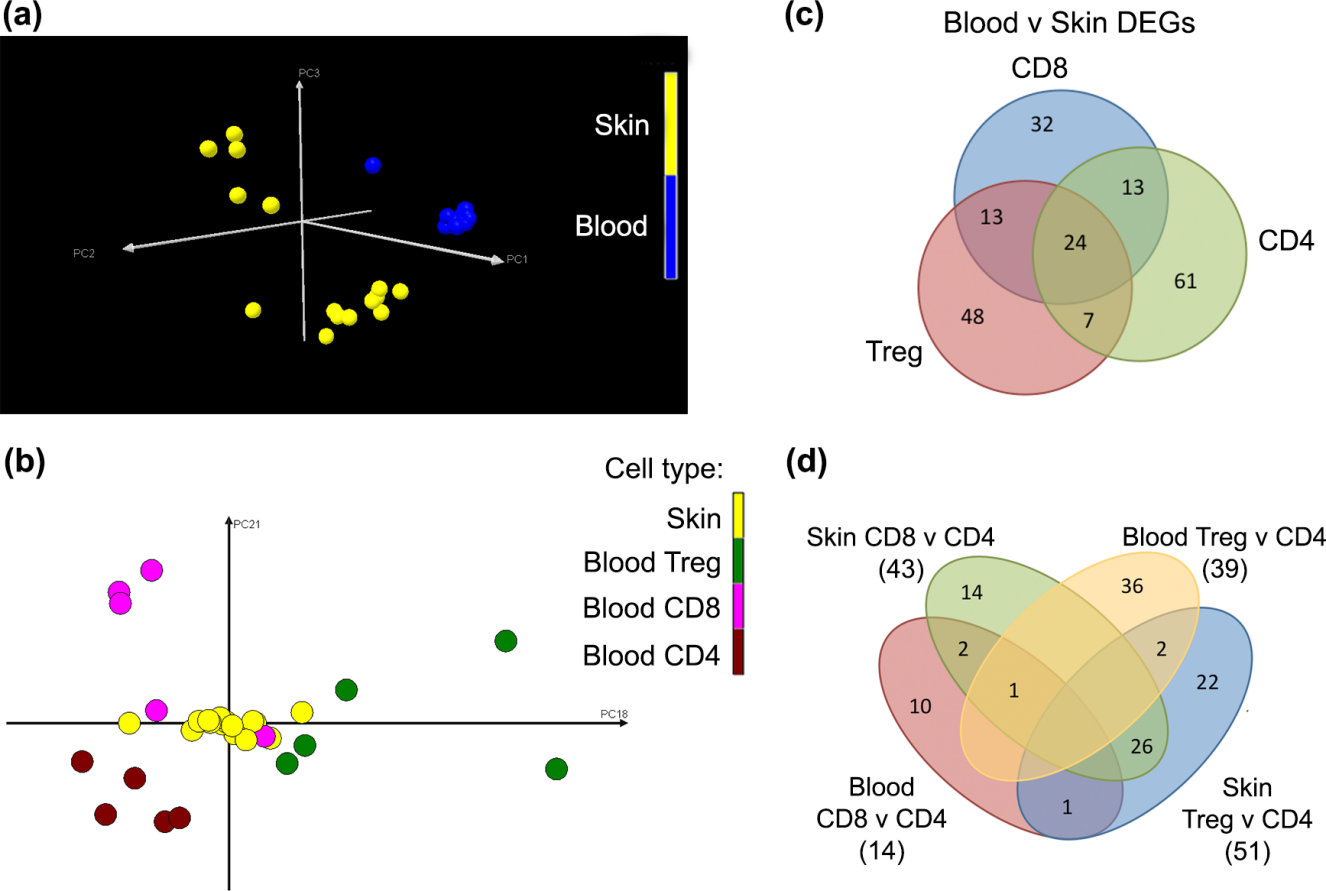
T cells in human skin are transcriptionally distinct from skin-tropic T cells in the blood. Principal component analysis of gene expression of sorted skin and blood CD4^+^, CD8^+^ and regulatory T cells. Graphs plotted showing (a) principal components 1,2 and 3 and (b) principal components 18 and 21. Each symbol represents one array; 5 arrays/cell type. (c) Venn diagram showing overlap of the significantly differentially expressed genes (DEGs, listed in S3 Table; P≤0.05) in pairwise comparison between skin and blood CD4^+^, CD8^+^ and regulatory T cells using RUVinv analysis. (d) Venn diagram of DEGs between T cell lineages in blood and skin (listed in S4 Table; P≤0.05) showing overlaps between categories. Numbers in brackets indicate total number of DEGs from each pairwise comparison. Treg, regulatory T cells.

We identified and isolated skin-homing memory T cells from human blood and skin based on the expression of CLA (Fig 1). The proportion of CLA^+^ memory T cells (CD8^+^, T_reg_ and CD4^+^) present in our samples was consistent with previous studies, constituting 80–90% of CD3^+^ cells in the skin, but only ~15% of CD3^+^ cells in the blood [1]. Gene expression profiles of these CD8^+^, CD4^+^ and T_reg_ CLA^+^ memory T cells in the skin and blood were examined through the use of microarray gene chips. Principal component analysis (PCA) was used to delineate variations in the transcriptional data. The PCA showed that the transcriptomes of the memory T cells segregated according to the tissue of origin on PC1 and 2 (73.4%; Fig 2A), whereas the variations due to T cell lineage were less prominent, as represented by PC18 and PC21 in blood (1.15%; Fig 2B). These results suggested that the magnitude of transcriptional dissimilarity between skin and blood T cells outweighed the gene expression differences between CD8^+^, T_reg_ and CD4^+^ T cells.

To determine which genes contributed to the transcriptional dissimilarities, we first performed pairwise comparisons between each skin T_reg_, CD4^+^ and CD8^+^ group and their blood equivalent. We found 80–100 significantly differentially expressed genes (DEGs) in each case (for complete lists see S3 Table). There was substantial overlap in DEGs, with a shared group of 24 genes that were differentially expressed in all three T cell types (Fig 2C and Table 1). As blood CLA^+^ T cells are known to be CD69^-^ [1], the significant upregulation of *CD69* (Table 1) provided some reassurance that cross-contamination between skin and blood T cells was minimal.

**Table 1:** Transcriptional signature of T cells resident in human skin.

Gene symbol	Description	Reg	Average log_2_FC
ATF3	Activating transcription factor 3	⬆	-2.21
CD69	CD69 molecule (early activation antigen)	⬆	-1.86
FOS	FBJ murine osteosarcoma viral oncogene homolog	⬆	-3.28
GLA	Galactosidase, alpha	⬆	-2.26
HSPH1	Heat shock 105kDa/110kDa protein 1	⬆	-4.04
NR4A1	Nuclear receptor subfamily 4, group A, member 1	⬆	-3.96
NR4A2	Nuclear receptor subfamily 4, group A, member 2	⬆	-2.37
PPP1R15A	Protein phosphatase 1, regulatory subunit 15A	⬆	-2.89
PTGER4	Prostaglandin E receptor 4 (subtype EP4)	⬆	-2.09
RASD1	RAS, dexamethasone-induced 1	⬆	-3.73
ASB3	Ankyrin repeat and SOCS box containing 3	⬇	1.72
ATP9B	ATPase, class II, type 9B	⬇	2.61
FAM160B1	Family with sequence similarity 160, member B1	⬇	2.49
KLF13	Kruppel-like factor 13	⬇	2.03
LEO1	LEO1 homolog, Paf1/RNA polymerase II complex component	⬇	1.74
MASP2	Mannan-binding lectin serine peptidase 2	⬇	2.92
P2RY8	Purinergic receptor P2Y, G-protein coupled, 8	⬇	2.26
RARRES3	Retinoid acid receptor responder (tazarotene induced) 3	⬇	2.01
RNF10	Ring finger protein 10	⬇	2.02
SEC61A2	Sec61 translocon alpha 2 subunit	⬇	2.07
TENM1	Teneurin transmembrane protein 1	⬇	2.00
TPP2	Tripeptidyl peptidase II	⬇	1.90
TSN	Translin	⬇	2.20
TSR2	TSR2, 20S rRNA accumulation, homolog (S. cerevisiae)	⬇	1.85

Shared differentially expressed genes amongst CD4^+^, CD8^+^ and regulatory T cells from normal human skin compared to skin-tropic blood T cells (overlapping region Fig 2C). Log_2_FC = Log_2_Fold-Change; negative values indicate higher expression in skin T cells and positive values indicate higher expression in blood T cells. Reg = Up- or downregulated in skin compared to blood T cells. P≤0.05 after multiple testing correction for all genes shown.

We then compared CD8^+^ to CD4^+^ T cells and T_reg_ to CD4^+^ T cells in skin and, separately, in blood to determine if there were also shared DEGs between T cell lineages independent of tissue origin. In contrast to the previous analysis between skin and blood T cells, there were ~40 DEGs identified in these pairwise comparisons (S4 Table), and these DEGs rarely overlapped between categories (Fig 2D). The difference in the number of DEGs identified between comparing cells from different sources and lineages reaffirmed the PCA findings, in that the environment the T cells are in greatly influences their transcriptional profiles, with some contributions from their developmental programs.

### Validation of microarray data using quantitative real-time PCR

**Fig 3:**
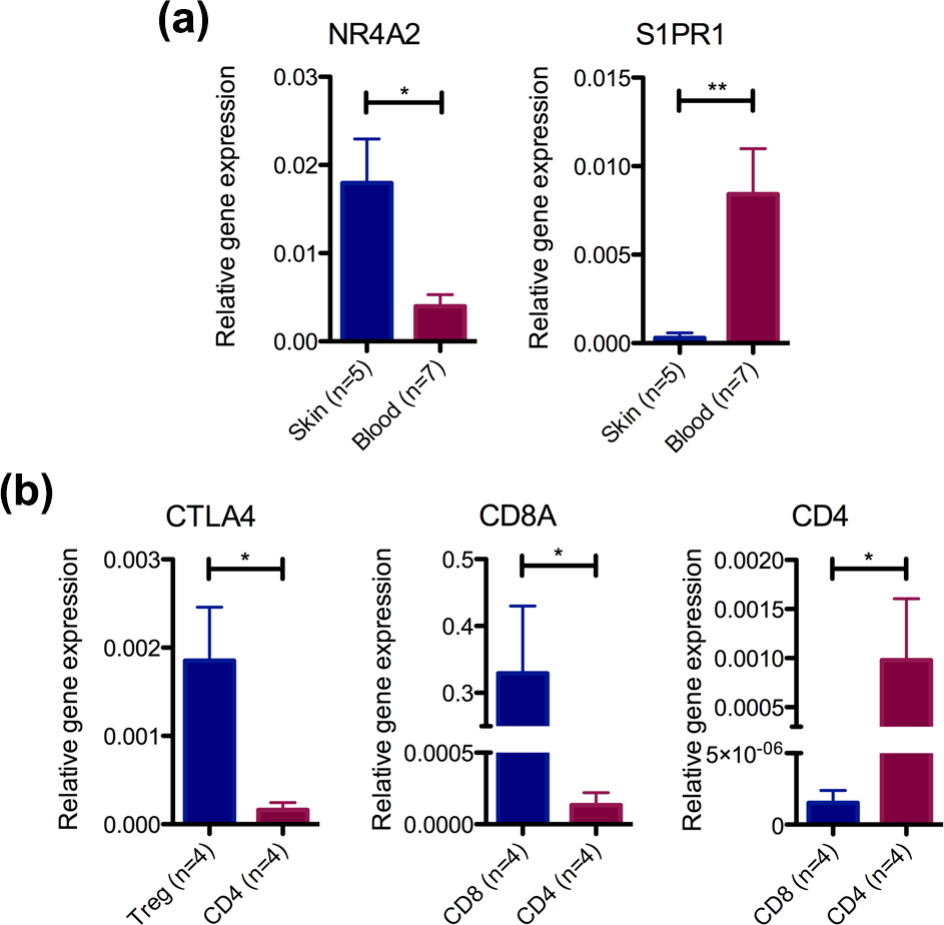
Skin and blood T cell subsets demonstrate differential gene expression by quantitative PCR. (a) qPCR was performed for selected genes differentially expressed between skin- and blood-derived T cells (combined CD8^+^, CD4^+^ and regulatory T cells) as identified on microarray analysis (Table 1 and S3 Table). Expression values were normalized to the geometric mean of two housekeeping genes. (b) Expression of lineage-related genes in the CD8^+^, CD4^+^ and regulatory T cells (combined blood and skin samples). *P≤0.05; **P≤0.01 using two-tailed Mann-Whitney U-test.

We sought to validate the DEGs identified in the microarray data analysis using qPCR. Due to limited specimen availability, we could only test a few DEGs in the remaining samples from each T cell subset. In concordance with the microarray data, T cells derived from skin showed higher expression of *NR4A2* compared to those from blood, whereas *S1PR1* expression was higher in T cells from blood than skin (Fig 3A). In the limited number of samples tested, *CCR7* showed a trend of increased expression in CD8^+^ T cells from blood compared to skin (Graph A in S1 Fig). Different T cell subsets from the skin and blood also showed elevated expression of their respective markers, *CTLA4*, *CD8A* and *CD4* (Fig 3B and Graph B in S1 Fig).

### Functional analysis suggests that skin T cells are active and responding to stress signals

**Fig 4:**
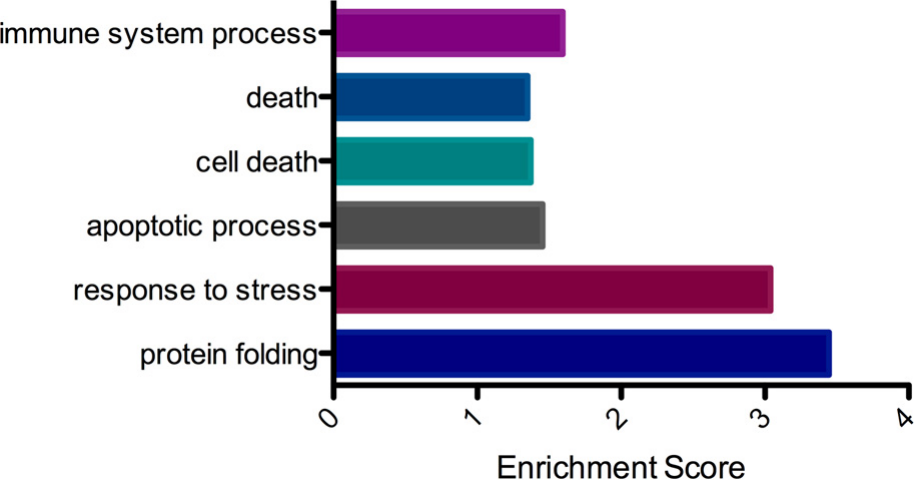
Skin T cells upregulate genes in immune system and stress response processes. Gene ontology analysis of transcripts upregulated in skin compared to blood T cells. Significantly overrepresented biological processes (PANTHER GO-Slim annotation; P≤0.05) in skin T cells shown plotted against Enrichment Score (calculated as -log_10_p-value).

Next, gene ontology (GO) terms were assigned to the genes that were upregulated and downregulated in skin T cells compared to blood counterparts, so as to identify their functions. GO terms were successfully matched to 75 of the 81 genes that were upregulated in skin T cells compared to blood T cells. Subsequent statistical analysis of GO terms enrichment showed that several biological processes were significantly overrepresented: protein folding, response to stress, apoptotic process, cell death, death and immune system process (Fig 4; for detailed results see S5 Table). Conversely, amongst the 115 genes that were upregulated in the blood T cells compared to skin, there were no significantly overrepresented biological processes found (data not shown). These results suggested that the skin T cells were poised to respond to danger signals and were in a more activated state than their blood equivalents.

**Fig 5:**
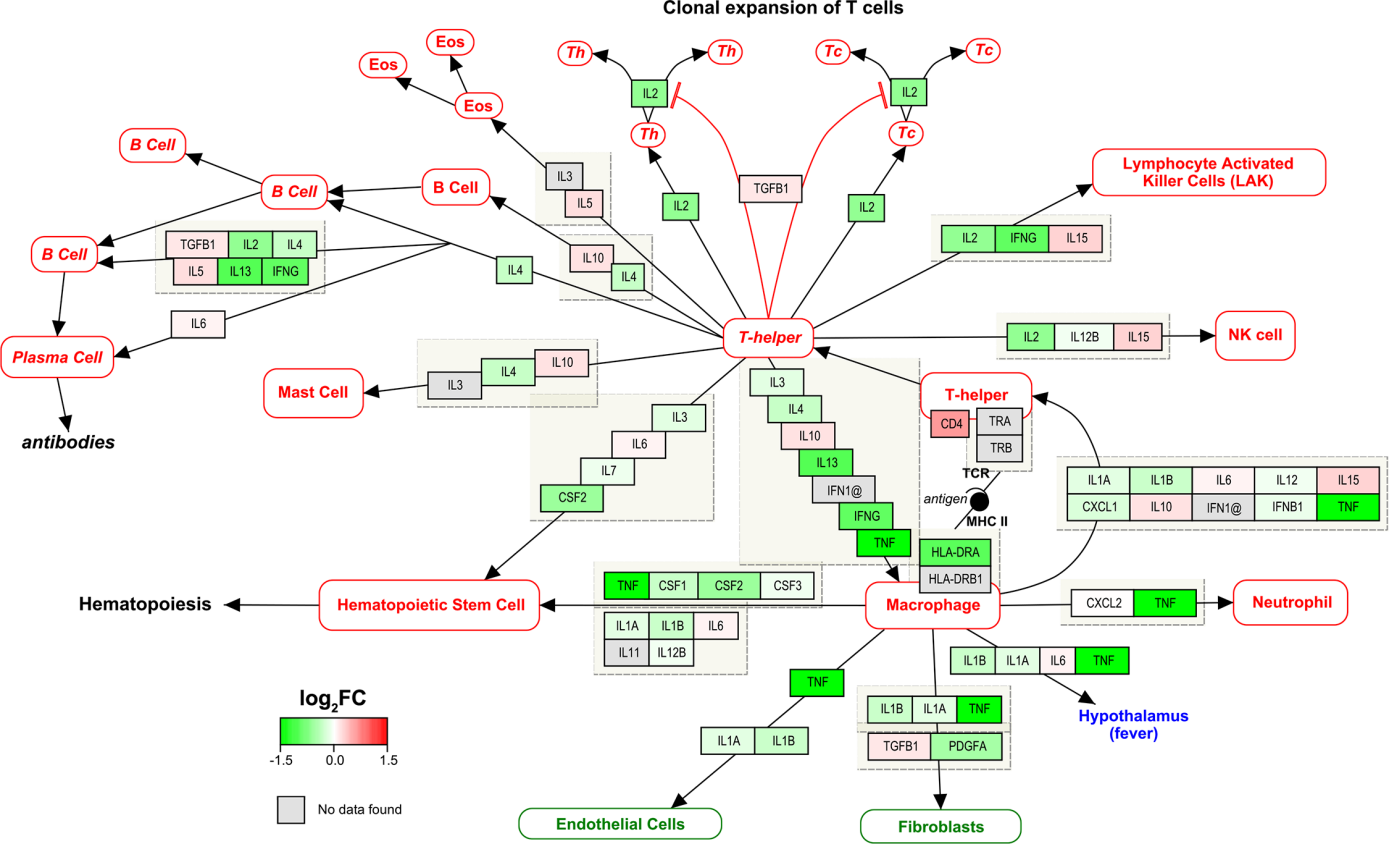
CD4^+^ T cells in the skin are transcriptionally active for cytokine and inflammatory response genes. Graphic representation of relative gene expression between skin CD4^+^ T cells and skin-tropic T cells derived from blood. Cytokines and inflammatory response pathway (WP530) depicted using PathVisio v3.2.1 and WikiPathways. Red-green colour bar denotes magnitude of log_2_fold-change (green = upregulated in skin, red = upregulated in blood). Similar results were found in CD8^+^ T cells and for regulatory T cells (data not shown).

**Fig 6:**
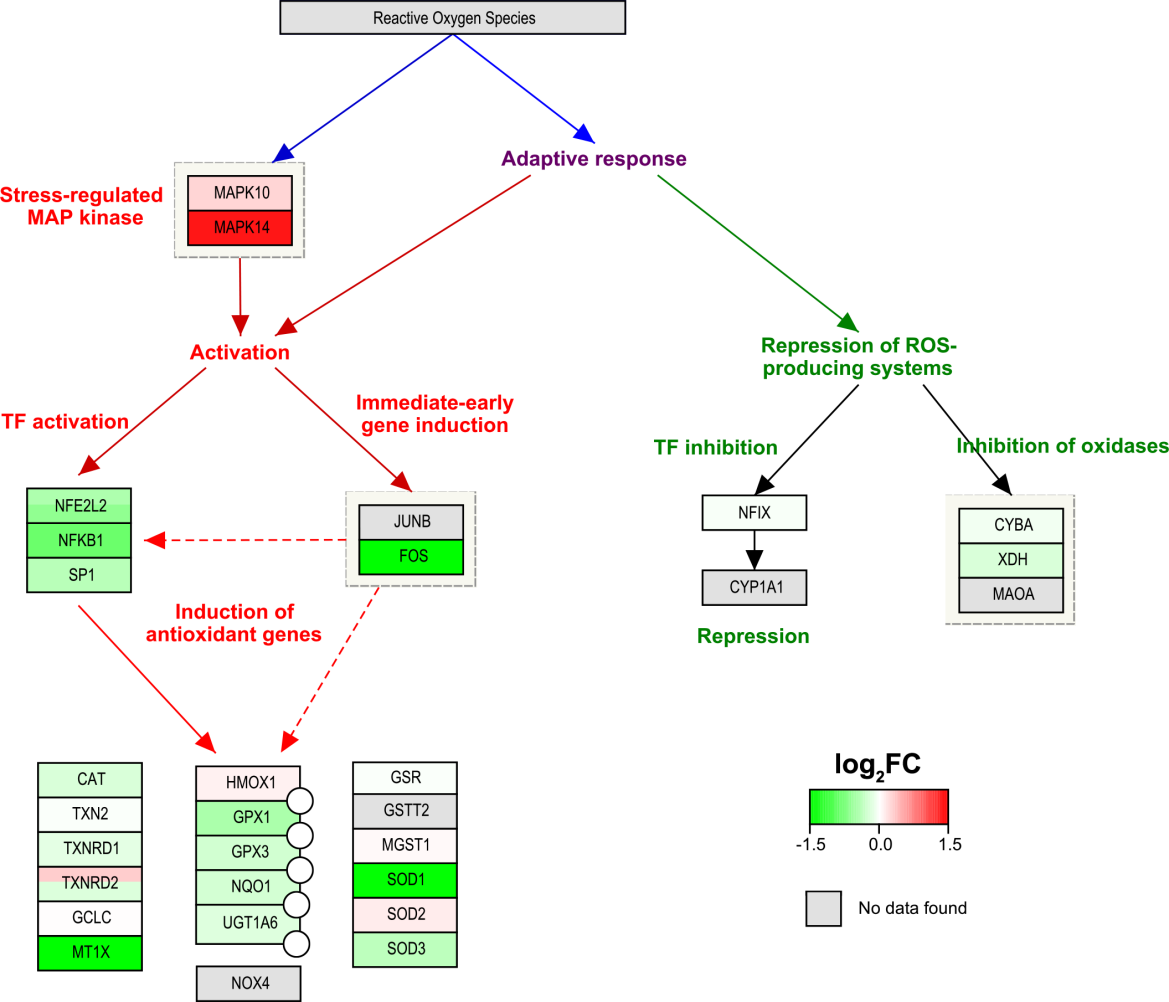
CD8^+^ T cells in the skin overexpress genes in the oxidative stress pathway. Graphic representation of relative gene expression between skin and blood CLA^+^ CD8^+^ T cells in the Oxidative Stress pathway (WP408) using PathVisio 3.2.1 and WikiPathways. Similar results were found for CD4^+^ T cells and for regulatory T cells (data not shown). Red-green colour bar denotes magnitude of log_2_fold-change (green = upregulated in skin, red = upregulated in blood). TF = transcription factor.

To investigate how the products of these gene transcripts might act within the cells, we performed pathway analysis on the T cell subsets by mapping our microarray results to relevant biological pathways found in the WikiPathways repository [26]. This pathway analysis was intended to provide a broad visual overview of the transcripts involved in a biological process, even though not all genes were necessarily significantly differentially expressed by our earlier stringent criteria. We found that the expression of genes involved in the Cytokines and Inflammatory Response pathway [29] (WP530) was skewed toward skin T cells (Fig 5). Similarly, many of the genes in the Oxidative Stress pathway [30] (WP408) were upregulated in skin T cells (Fig 6). Combined, the increased transcription of inflammatory cytokines and stress response-related genes provides additional evidence that skin T cells are indeed in a highly activated state.

### Transcriptional profiles of skin T cells are significantly enriched for T_RM_ core signature genes

**Fig 7:**
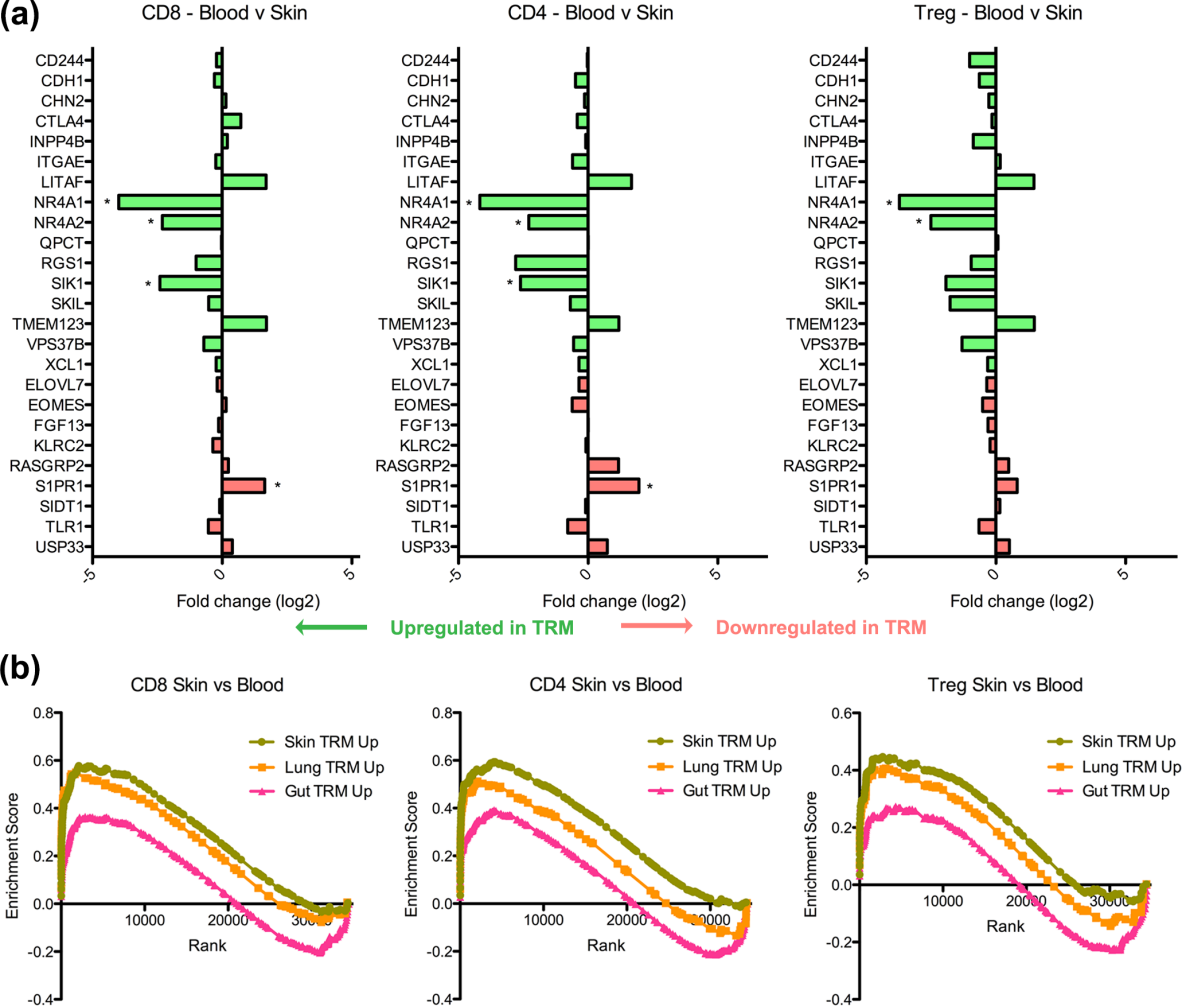
Human skin T cell transcription profiles are enriched for signature resident memory T cell genes defined in the mouse. (a) The fold change of various genes in the murine skin T_RM_ core signature in human blood versus skin CD4^+^, CD8^+^ and T_reg_ cells on microarray analysis. Green (upregulated in T_RM_) and red arrows (downregulated in T_RM_) below indicate the expected direction of expression in T_RM_. Asterisks indicate significantly differentially expressed genes (P≤0.05; n = 5 arrays per cell type). (b) Enrichment scores for the various skin T cell types following Gene Set Enrichment Analysis using gene set lists containing lung, gut and skin T_RM_ gene signatures. All gene sets shown are significantly enriched at false discovery rate <25%.

Finally, we sought to compare the human skin T cell transcriptome to the core signature of CD8^+^ T_RM_ as established in the mouse [7]. The various skin T cell types loosely conformed to the gene expression pattern of the murine T_RM_ core signature, with all significant DEGs corresponding exactly to the T_RM_ profile (Fig 7A). Further analysis using GSEA was performed on the genes typically up- and downregulated in murine lung, skin and gut T_RM_ compared to circulating T_EM_ and T_CM_ (S2 Table). Surprisingly, we found that the transcriptional profiles of human skin CD4^+^ T cells, T_reg_ and CD8^+^CD103^-^ T cells were all individually significantly enriched for genes upregulated in murine T_RM_ compared to the human skin-tropic T cells in blood (Fig 7B). In keeping with this result, the T cells derived from human blood were significantly enriched for genes downregulated in murine T_RM_ (S2 Fig).

We were interested in ascertaining which genes in our T cell samples were most important in accounting for their similarity to the murine T_RM_ gene signatures. To identify the subset of genes that had the greatest contribution to the enrichment scores [27], we performed a leading edge analysis on the GSEA datasets. We found that there were many shared genes between the leading edge subsets for CD8^+^CD103^-^, T_reg_ and CD4^+^ T cells (Table 2; for complete leading edge subset lists see S6 Table). Among these genes, we identified aryl hydrocarbon receptor (*AHR*), which is a critical determinant in enabling T cells to persist in the skin [31]. Taken together, our results suggest that while in the skin, CD4^+^, T_reg_ and CD8^+^CD103^-^ T cells acquire a similar gene expression profile to that of T_RM_, thereby potentially allowing them to reside long-term within skin.

**Table 2:** Shared genes of human skin T cells and murine T_RM_ signature with greatest contribution to the gene set enrichment.

Upregulated in skin		Downregulated in skin	
Gene Symbol	Description	Gene symbol	Description
AHR	Aryl hydrocarbon receptor	ARAP2	ArfGAP with RhoGAP domain, ankyrin repeat and PH domain 2
ANXA1	Annexin A1	ARL2BP	ADP-ribosylation factor-like 2 binding protein
CD69	CD69 molecule	BIN2	Bridging integrator 2
DUSP1	Dual specificity phosphatase 1	CCL5	Chemokine (C-C motif) ligand 5
DUSP6	Dual specificity phosphatase 6	DGKA	Diacylglycerol kinase, alpha 80kDa
EGR1	Early growth response 1	GIMAP4	GTPase, IMAP family member 4
EGR2	Early growth response 2	GIMAP7	GTPase, IMAP family member 7
ELL2	Elongation factor, RNA polymerase II, 2	IFIT3	Interferon-induced protein with tetratricopeptide repeats 3
FOS	FBJ murine osteosarcoma viral oncogene homolog	ITGB1	Integrin, beta 1
FOSB	FBJ murine osteosarcoma viral oncogene homolog B	KLF3	Kruppel-like factor 3
GADD45B	Growth arrest and DNA-damage-inducible, beta	KLHL24	Kelch-like family member 24
GEM	GTP binding protein overexpressed in skeletal muscle	PYHIN1	Pyrin and HIN domain family, member 1
HSPA1A	Heat shock 70kDa protein 1A	RASA3	RAS p21 protein activator 3
LMNA	Lamin A/C	RASGRP2	RAS guanyl releasing protein 2
NFKBID	Nucler factor of kappa light polypeptide gene enhancer in B-cells inhibitor, delta	S1PR1	Sphingosine-1-phosphate receptor 1
NR4A1	Nuclear receptor subfamily 4, group A, member 1	SH2D1A	SH2 domain containing 1A
NR4A3	Nuclear receptor subfamily 4, group A, member 3	STK38	Serine/threonine kinase 38
PHLDA1	Pleckstrin homology-like domain, family A, member 1	USP33	Ubiquitin specific peptidase 33
PPP1R15A	Protein phosphatase 1, regulatory subunit 15A		
RGS1	Receptor of G-protein signaling 1		
RGS2	Receptor of G-protein signaling 2		
SIK1	Salt-inducible kinase 1		
STYK1	Serine/threonine/tyrosine kinase 1		
TNF	Tumor necrosis factor		
TNFRSF9	Tumor necrosis factor receptor superfamily, member 9		
TNFSF9	Tumor necrosis factor (ligand) superfamily, member 9		

Leading edge analysis was performed to determine which genes contributed most to the enrichment scores shown in Fig 7 and S2 Fig for the gene sets listing genes upregulated and downregulated in murine skin resident memory T cells (skin T_RM_). Shared genes common to the leading edge subsets for CD4^+^, CD8^+^ and regulatory T cells are shown.

## Discussion

Normal human skin contains both T_RM_ and T_RCM_. The best-characterized T_RM_ are intraepithelial CD8^+^CD103^+^ T cells, although recently CD4^+^ T_RM_ have also been described, and the necessity of CD103 expression has been disputed [13,14,32,33]. In this paper, we analyzed the transcriptomes of CD8^+^, T_reg_ and non-T_reg_ CD4^+^ T cells bearing the skin-homing marker CLA in the blood to the same populations in the skin. In essence, we compared groups of T cells containing both T_RM_ and T_RCM_ (skin) to groups composed exclusively of T_RCM_ (blood). We showed that skin-derived T cells possess a gene signature different from blood-derived T cells and this signature is largely conserved in both humans and mice.

Several key genes that were differentially expressed between skin and blood T cells are associated with tissue persistence and cell activation status. For example, *S1PR1*, which mediates T cell egress [34], was significantly downregulated in CD4^+^ and CD8^+^CD103^-^ cutaneous T cells, although fell short of significance in cutaneous T_reg_. In addition, skin T cells displayed higher expression of the early activation marker *CD69* and upregulated genes involved in the stress response and immune process pathways. Further comparison of human skin T cells to the common transcription profile of murine CD8^+^CD103^+^ T_RM_ found in various murine tissues revealed that the expression pattern of murine T_RM_ signature genes was largely conserved in human skin CD8^+^CD103^-^, T_reg_ and CD4^+^ cells, and that a shared group of genes accounted for their likeness to murine T_RM_, including potential lineage transcription factors associated with murine T_RM_.

There are a number of ways to interpret our findings. The first is that the differences we observed between skin and blood T cells could be, wholly or partially, due to changes induced by the different microenvironments. It may be that gene expression is altered so that circulating T cells can enter and take up residence in the skin, with the initial changes then triggering downstream effects on cell behavior and effector function [34]. Moreover, since healthy skin contains numerous commensal microbes [35] extending even into the dermis [36], there would conceivably be a constant supply of antigens to stimulate T cell activation. These stimuli are not usually present in the circulation, which could result in a difference in activation status. The implication of this model is that T cells leaving the skin would eventually lose the skin-specific gene expression signature. Interestingly, *in vivo* tracking of skin T_RCM_ in the Kaede transgenic murine model demonstrated that T_RCM_ displayed the same surface phenotype for days after leaving the skin [14]. However, as surface markers may not accurately reflect changes at the gene expression level, further investigation matching the gene expression in T_RCM_ derived from blood and skin is required to definitively exclude this possibility.

Another possible interpretation is that each of the CD8^+^CD103^-^, T_reg_ and CD4^+^ T cell groups in human skin contain substantial amounts of T_RM_, or cells that are developing into T_RM_, thereby accounting for the differences in the gene expression profiles. This interpretation would certainly be compatible with the situation found in patients with cutaneous T cell lymphoma (CTCL) treated with alemtuzumab, where a similarly diverse range of T cells were spared from depletion and remained in the skin post-treatment [13]. If this is the case, the DEGs identified between the skin and blood T cells would be representative of the transcription profile of the T_RM_ population within skin. Taken together with the results from the pathway, gene ontology and principal component analyses, our findings imply that significant functional distinctions exist between T_RM_ and T_RCM_ within a T cell lineage [4,37], which are potentially as important as the differences between the various T cell lineages themselves.

Finally, an intriguing hypothesis that can also be drawn from our studies, is that T_RM_ and T_RCM_ are two ends of a spectrum of extralymphoid memory T cells, with a number of intermediate phenotypes that could reside in skin for varying periods of time. Their time in residency may even depend on their strength of expression of the T_RM_ core signature. The existence of intermediate phenotypes would explain the observed expression of T_RM_-associated genes even in cutaneous T cells that lack defining features of T_RM_, such as CD103. Furthermore, this theory is supported by the recent discovery that T_RCM_ in CTCL patients could be further delineated into two major subgroups with differing rates of depletion from the skin by alemtuzumab [32]. As the duration of observation in previous studies has been limited by logistical and technical factors (up to 6 months for mouse experiments and ~6 weeks in alemtuzumab patients [32,38]), it is unknown whether some of the T cells that persisted early on in the skin might eventually migrate out into the circulation. Importantly, the abovementioned hypotheses need not be mutually exclusive, as features unique to the skin microenvironment may both recruit T cells as well as encourage their development along the T_RCM_-T_RM_ continuum [39].

In summary, our report provides evidence that CD8^+^CD103^-^, T_reg_ and CD4^+^ T_RM_ may exist in healthy human skin, sharing a common skin-associated gene expression signature. The distinction between T_RCM_ and T_RM_ will provide new insights into T cell biology, with potential transcriptional and functional consequences on par with the CD4^+^ and CD8^+^ T cell lineages.

## Supporting Information

S1 Fig Validation of microarray results using quantitative real-time PCR. (a) PCR was performed to examine CCR7 expression in sorted skin- and blood-derived CD8^+^ T cells. (b) Expression of CD4 in skin-derived CD8^+^ and CD4^+^ T cells as determined by PCR.
(PDF)

S2 Fig Skin-tropic T cells in the blood are enriched for genes downregulated in resident memory T cells. Results of Gene Set Enrichment Analysis using gene set lists of genes downregulated in lung, gut and skin resident memory T cells (T_RM_). Negative enrichment scores indicate that gene sets are enriched in blood compared to skin samples. All gene sets shown are significantly enriched at False Discovery Rate <25%. N = 5 arrays per cell type.
(PDF)

S1 Table T cell populations sorted from blood and skin for microarray. Surface markers were used to identify and sort live T cell populations from skin and blood for RNA extraction. For each of the 6 cell types, 5 biological replicates were obtained.
(PDF)

S2 Table Gene sets used for Gene Set Enrichment Analysis. Gene sets contain lists of genes, compiled in Illumina probe ID format, that are typically up- or downregulated in resident memory T cells (T_RM_) from lung, skin and gut.
(PDF)

S3 Table Significantly differentially expressed genes between blood and skin T cells. Significantly differentially expressed genes (DEGs) identified after pairwise comparison of microarray results with the RUVinv statistical method. Log_2_Fold-Change (log_2_FC) cutoff of 1.5 used. P<0.05 after multiple testing correction for all genes shown. Bold = differentially expressed genes shared between all 3 groups.
(PDF)

S4 Table Significantly differentially expressed genes between T cell lineages in blood and in skin. Significantly differentially expressed genes identified after pairwise comparison of microarray results with the RUVinv statistical method. Log_2_Fold-Change (log_2_FC) cutoff of 1.5 used. P<0.05 after multiple testing correction for all genes shown. Bold = common between blood and skin CD8 versus CD4 T cells. Bold italicized = common between blood and skin Treg versus CD4 T cells.
(PDF)

S5 Table Gene ontology (GO) analysis of differentially expressed genes upregulated in skin T cells compared to blood T cells. Data obtained from PANTHER version 10.0 Overrepresentation Test (release 20150430) using PANTHER GO-Slim Biological Process annotation data set. P-values are adjusted for multiple testing with the Bonferroni method.
(PDF)

S6 Table Results of leading edge analysis of Gene Set Enrichment Analysis. Leading edge analysis was performed to determine which genes in the various skin T cell types contributed most to the enrichment score for the gene sets pertaining to skin resident memory T cells (T_RM_), i.e. gene sets containing the genes upregulated in skin T_RM_ and downregulated in skin T_RM_. Treg = regulatory T cells. Bold = shared leading edge subset genes between the 3 groups.
(PDF)
