# Supplementary material for: Transcriptional Analysis of T Cells Resident in Human Skin
Source: PLoS One. 2016 Jan 29;11(1):e0148351. doi: 10.1371/journal.pone.0148351 (PMC4732610; doi:10.1371/journal.pone.0148351)
Supplement: S1 Fig — (a) PCR was performed to examine CCR7 expression in sorted skin- and blood-derived CD8+ T cells. (b) Expression of CD4 in skin-derived CD8+ and CD4+ T cells as determined by PCR. (PDF) [file pone.0148351.s001.pdf]

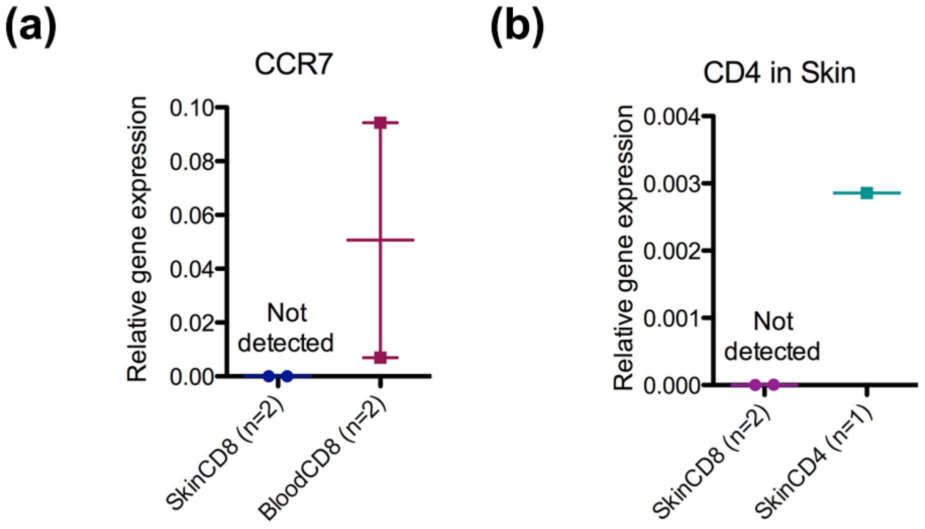

S1 Fig. Validation of microarray results using quantitative real-time PCR.

(a) PCR was performed to examine CCR7 expression in sorted skin- and blood-derived $CD8^+$ T cells. (b) Expression of CD4 in skin-derived $CD8^+$ and $CD4^+$ T cells as determined by PCR.
