# Supplementary material for: Transcriptional Analysis of T Cells Resident in Human Skin
Source: PLoS One. 2016 Jan 29;11(1):e0148351. doi: 10.1371/journal.pone.0148351 (PMC4732610; doi:10.1371/journal.pone.0148351)
Supplement: S2 Fig — Results of Gene Set Enrichment Analysis using gene set lists of genes downregulated in lung, gut and skin resident memory T cells (TRM). Negative enrichment scores indicate that gene sets are enriched in blood compared to skin samples. All gene sets shown are significantly enriched at False Discovery Rate <25%. N = 5 arrays per cell type. (PDF) [file pone.0148351.s002.pdf]

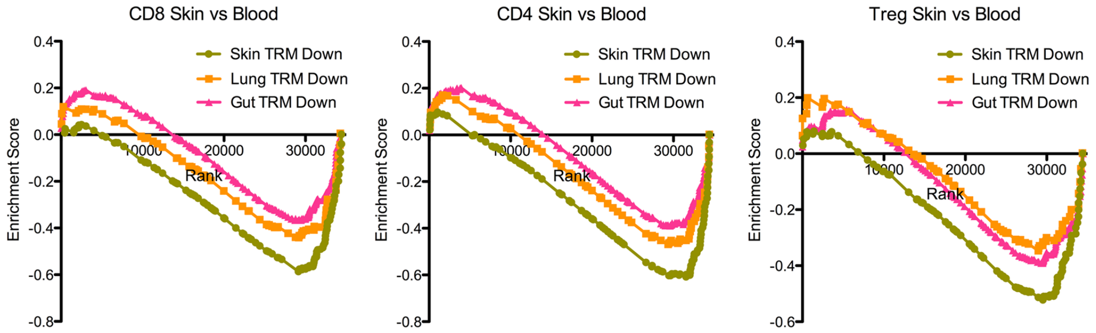

S2 Fig. Skin-tropic T cells in the blood are enriched for genes downregulated in resident memory T cells. Results of Gene Set Enrichment Analysis using gene set lists of genes downregulated in lung, gut and skin resident memory T cells (T_{RM}). Negative enrichment scores indicate that gene sets are enriched in blood compared to skin samples. All gene sets shown are significantly enriched at False Discovery Rate <25%. N=5 arrays per cell type.
