# Supplementary material for: Transcriptional Analysis of T Cells Resident in Human Skin
Source: PLoS One. 2016 Jan 29;11(1):e0148351. doi: 10.1371/journal.pone.0148351 (PMC4732610; doi:10.1371/journal.pone.0148351)
Supplement: S1 Table — Surface markers were used to identify and sort live T cell populations from skin and blood for RNA extraction. For each of the 6 cell types, 5 biological replicates were obtained. (PDF) [file pone.0148351.s003.pdf]

S1 Table. T cell populations sorted from blood and skin for microarray.

Source	Description	Gating Strategy
Blood	Skin-tropic memory T cytotoxic cells	$CD3^+$ CLA^+ $CD45RO^+$ $CD8^+$
Blood	Skin-tropic memory T helper cells	$CD3^+$ CLA^+ $CD45RO^+$ $CD4^+$ $CD127_{hi}$
Blood	Skin-tropic T regulatory cells	$CD3^+$ CLA^+ $CD4^+$ $CD25_{hi}$ $CD127_{lo}$
Skin	Skin-tropic memory T cytotoxic cells	$CD45^+$ $CD3^+$ CLA^+ $CD8^+$ $CD103^-$
Skin	Skin-tropic memory T helper cells	$CD45^+$ $CD3^+$ CLA^+ $CD4^+$ $CD127_{hi}$
Skin	Skin-tropic T regulatory cells	$CD45^+$ $CD3^+$ CLA^+ $CD4^+$ $CD25_{hi}$ $CD127_{lo}$

Surface markers were used to identify and sort live T cell populations from skin and blood for RNA extraction. For each of the 6 cell types, 5 biological replicates were obtained.

CD45 was used as a marker in the skin samples to eliminate cells of a non-haematopoietic origin. The memory marker CD45RO was used in the blood samples to ensure that only $CD4^+$ and $CD8^+$ T cells with a memory phenotype were isolated. As previous studies have indicated that CLA^+ regulatory T cells in blood and CLA^+ skin T cells almost universally express CD45RO [1,2], CD45RO was not included as a marker for these subsets.

References

1. Clark RA, Chong B, Mirchandani N, Brinster NK, Yamanaka K, et al. (2006) The vast majority of CLA+ T cells are resident in normal skin. Journal of Immunology 176: 4431-4439.

2. Hirahara K, Liu L, Clark RA, Yamanaka K, Fuhlbrigge RC, et al. (2006) The majority of human peripheral blood CD4+CD25highFoxp3+ regulatory T cells bear functional skin-homing receptors. Journal of Immunology 177: 4488-4494.
