# Supplementary material for: Transcriptional Analysis of T Cells Resident in Human Skin
Source: PLoS One. 2016 Jan 29;11(1):e0148351. doi: 10.1371/journal.pone.0148351 (PMC4732610; doi:10.1371/journal.pone.0148351)
Supplement: S2 Table — Gene sets contain lists of genes, compiled in Illumina probe ID format, that are typically up- or downregulated in resident memory T cells (TRM) from lung, skin and gut. (PDF) [file pone.0148351.s004.pdf]

S2 Table. Gene sets used for Gene Set Enrichment Analysis.

Skin T_{RM}		Lung T_{RM}		Gut T_{RM}	
**Up**	**Down**	**Up**	**Down**	**Up**	**Down**
ILMN_1730710	ILMN_2078599	ILMN_1713751	ILMN_1711490	ILMN_1755658	ILMN_2184184
ILMN_1733259	ILMN_1767541	ILMN_1682225	ILMN_1656159	ILMN_2152711	ILMN_2409167
ILMN_2162799	ILMN_3244434	ILMN_1788481	ILMN_2124155	ILMN_1670912	ILMN_1755937
ILMN_1788462	ILMN_1709360	ILMN_1778723	ILMN_1658884	ILMN_1713751	ILMN_1711899
ILMN_3227060	ILMN_1711490	ILMN_1796663	ILMN_1759628	ILMN_1682225	ILMN_2362122
ILMN_1667994	ILMN_1664016	ILMN_1655821	ILMN_1783304	ILMN_1788481	ILMN_1667086
ILMN_2184184	ILMN_1755391	ILMN_1702534	ILMN_1654322	ILMN_1673119	ILMN_1711490
ILMN_2409167	ILMN_3248700	ILMN_2233783	ILMN_1813399	ILMN_2129015	ILMN_2043816
ILMN_1755937	ILMN_1656159	ILMN_1770940	ILMN_1750599	ILMN_2162799	ILMN_2124155
ILMN_1711899	ILMN_1759628	ILMN_1774110	ILMN_2401933	ILMN_2214232	ILMN_1658884
ILMN_2120022	ILMN_1783304	ILMN_2292187	ILMN_2088437	ILMN_1681301	ILMN_1759628
ILMN_1680465	ILMN_1654322	ILMN_2403237	ILMN_1745788	ILMN_1690465	ILMN_1783304
ILMN_1655821	ILMN_1726342	ILMN_1732716	ILMN_1760554	ILMN_1733674	ILMN_1654322
ILMN_2086965	ILMN_2098126	ILMN_1738207	ILMN_1655521	ILMN_2108709	ILMN_1702609
ILMN_1702534	ILMN_1773352	ILMN_1703074	ILMN_1740213	ILMN_1690884	ILMN_2082810
ILMN_1803429	ILMN_1800540	ILMN_2302947	ILMN_1760509	ILMN_1795826	ILMN_1696420
ILMN_2348788	ILMN_1698367	ILMN_1733402	ILMN_1773262	ILMN_1693896	ILMN_3185563
ILMN_1778625	ILMN_1673363	ILMN_2261627	ILMN_2212878	ILMN_1796663	ILMN_3262031
ILMN_2188333	ILMN_2413508	ILMN_2348905	ILMN_3204346	ILMN_1737314	ILMN_1655821
ILMN_1651316	ILMN_1760554	ILMN_1763487	ILMN_1794038	ILMN_1746053	ILMN_1716057
ILMN_1770940	ILMN_1661544	ILMN_1775304	ILMN_1655906	ILMN_1768534	ILMN_3232921
ILMN_1787212	ILMN_2319910	ILMN_1712959	ILMN_1770484	ILMN_2182704	ILMN_3188479
ILMN_1774110	ILMN_1661454	ILMN_1655930	ILMN_1754279	ILMN_1768194	ILMN_1777461
ILMN_2292187	ILMN_2319913	ILMN_2368773	ILMN_1668634	ILMN_1681644	ILMN_1658299
ILMN_2403237	ILMN_1752455	ILMN_1777261	ILMN_2331121	ILMN_1712718	ILMN_2276996
ILMN_1732716	ILMN_1692535	ILMN_1693009	ILMN_1660027	ILMN_1653001	ILMN_2376431
ILMN_1738207	ILMN_1740213	ILMN_1739397	ILMN_2382403	ILMN_1664316	ILMN_1669062
ILMN_2400407	ILMN_1760509	ILMN_1749448	ILMN_1653826	ILMN_2337386	ILMN_1771333
ILMN_1661852	ILMN_1773262	ILMN_1697228	ILMN_1690984	ILMN_1742001	ILMN_2356991
ILMN_1745318	ILMN_2212878	ILMN_1782230	ILMN_1671777	ILMN_1702534	ILMN_2061043
ILMN_2302947	ILMN_1794038	ILMN_1705587	ILMN_1780368	ILMN_2233783	ILMN_1698367
ILMN_1733402	ILMN_2331121	ILMN_1798977	ILMN_1721762	ILMN_2188333	ILMN_1736327
ILMN_1703123	ILMN_1660027	ILMN_1815054	ILMN_1769433	ILMN_1651316	ILMN_3177833
ILMN_2261627	ILMN_2382403	ILMN_1765941	ILMN_2254635	ILMN_1770940	ILMN_1714759
ILMN_2348905	ILMN_1653826	ILMN_1709085	ILMN_1730176	ILMN_1787212	ILMN_1656920
ILMN_1763487	ILMN_1690984	ILMN_2119320	ILMN_1753518	ILMN_1742779	ILMN_1674038
ILMN_1716875	ILMN_1671777	ILMN_1789074	ILMN_1735779	ILMN_1677376	ILMN_2088437
ILMN_2041161	ILMN_1748473	ILMN_1660436	ILMN_1735930	ILMN_1774110	ILMN_1745788
ILMN_1759327	ILMN_2329429	ILMN_1669927	ILMN_2048507	ILMN_2292187	ILMN_2246410
ILMN_3280238	ILMN_1757785	ILMN_1801246	ILMN_1670245	ILMN_2403237	ILMN_2320888
ILMN_3199483	ILMN_2133316	ILMN_1805750	ILMN_1768814	ILMN_1732716	ILMN_1801584
ILMN_1781285	ILMN_1776678	ILMN_2198878	ILMN_1719093	ILMN_1751615	ILMN_1655521
ILMN_1677466	ILMN_1711617	ILMN_3188500	ILMN_2213136	ILMN_2225735	ILMN_1752455
ILMN_2396020	ILMN_1806692	ILMN_1716234	ILMN_1679185	ILMN_1668582	ILMN_1791296
ILMN_1762899	ILMN_1664543	ILMN_1659913	ILMN_1671554	ILMN_1745318	ILMN_1740213
ILMN_1743199	ILMN_1701789	ILMN_1683927	ILMN_1681356	ILMN_1703123	ILMN_1801616
ILMN_1655930	ILMN_2239754	ILMN_1713934	ILMN_2276397	ILMN_2261627	ILMN_1765446
ILMN_3266606	ILMN_1721762	ILMN_1785527	ILMN_2375319	ILMN_2348905	ILMN_1760509
ILMN_3287239	ILMN_2342579	ILMN_1732452	ILMN_1794594	ILMN_1763487	ILMN_1731001
ILMN_2146761	ILMN_1691341	ILMN_1707631	ILMN_3235853	ILMN_2092041	ILMN_1904238
ILMN_2146766	ILMN_1747052	ILMN_1651958	ILMN_1653504	ILMN_1746864	ILMN_2104696
ILMN_1696302	ILMN_2254635	ILMN_2071809	ILMN_1659158	ILMN_3235928	ILMN_1773262
ILMN_1674080	ILMN_1730176	ILMN_2235851	ILMN_2258816	ILMN_3191227	ILMN_2212878
ILMN_1652797	ILMN_1753518	ILMN_2408566	ILMN_2343618	ILMN_1778717	ILMN_1761159
ILMN_1713266	ILMN_1723467	ILMN_2410145	ILMN_1755415	ILMN_1732039	ILMN_2041190
ILMN_2123743	ILMN_1714820	ILMN_1661178	ILMN_3229324	ILMN_3244638	ILMN_2202894
ILMN_1693009	ILMN_1754233	ILMN_2339955	ILMN_3305938	ILMN_2041161	ILMN_1728923

ILMN_2269380	ILMN_1784454	ILMN_1782305	ILMN_1702487	ILMN_1759327	ILMN_3204346
ILMN_2362581	ILMN_2244108	ILMN_1806725	ILMN_1853711	ILMN_3280238	ILMN_2331121
ILMN_1705111	ILMN_2383934	ILMN_3251550	ILMN_1795118	ILMN_3199483	ILMN_1660027
ILMN_1669523	ILMN_2175912	ILMN_1687978	ILMN_1774806	ILMN_2325008	ILMN_2382403
ILMN_1751607	ILMN_1735779	ILMN_2093343	ILMN_1678095	ILMN_2248589	ILMN_1653826
ILMN_1725175	ILMN_2048507	ILMN_1653026	ILMN_1789913	ILMN_1653047	ILMN_1690984
ILMN_1718977	ILMN_1670245	ILMN_1731589	ILMN_1707005	ILMN_1690844	ILMN_1671777
ILMN_1788802	ILMN_1678671	ILMN_2113049	ILMN_2367141	ILMN_2402416	ILMN_1687335
ILMN_1812759	ILMN_1768814	ILMN_1741727	ILMN_1710746	ILMN_1793770	ILMN_3284000
ILMN_2335813	ILMN_2124920	ILMN_2316844	ILMN_1683986	ILMN_1784665	ILMN_1740770
ILMN_3276095	ILMN_1658399	ILMN_1726913	ILMN_1676470	ILMN_1759175	ILMN_1661138
ILMN_2225135	ILMN_2213136	ILMN_1708537	ILMN_1677846	ILMN_1759488	ILMN_1806692
ILMN_1666545	ILMN_1679185	ILMN_1656011	ILMN_1731048	ILMN_2401878	ILMN_2389501
ILMN_1678968	ILMN_1671554	ILMN_1733538	ILMN_1654560	ILMN_2401873	ILMN_2389506
ILMN_2367883	ILMN_1675956	ILMN_1668559	ILMN_1741143	ILMN_1743199	ILMN_1811370
ILMN_1677092	ILMN_1669031	ILMN_1811890	ILMN_1723536	ILMN_1724984	ILMN_1801246
ILMN_1739397	ILMN_1663131	ILMN_1808226	ILMN_2306077	ILMN_1655930	ILMN_1728724
ILMN_1749448	ILMN_2114422	ILMN_2197365	ILMN_1688698	ILMN_1774779	ILMN_1781700
ILMN_1656818	ILMN_1681356	ILMN_1655347		ILMN_3246560	ILMN_1721762
ILMN_2092333	ILMN_3245773	ILMN_2206949		ILMN_1731231	ILMN_1769433
ILMN_1701947	ILMN_2093343	ILMN_1717639		ILMN_1703180	ILMN_1723467
ILMN_1723274	ILMN_1653026	ILMN_3235647		ILMN_1678965	ILMN_1714820
ILMN_1697228	ILMN_1733421	ILMN_2098119		ILMN_2392261	ILMN_1754233
ILMN_1782230	ILMN_1742026	ILMN_1736834		ILMN_1721559	ILMN_1784454
ILMN_1705587	ILMN_1669447	ILMN_1782938		ILMN_1775498	ILMN_2244108
ILMN_1798977	ILMN_2395981	ILMN_2317658		ILMN_1744508	ILMN_2383934
ILMN_1815054	ILMN_1654586	ILMN_1679041		ILMN_1725175	ILMN_2175912
ILMN_1765941	ILMN_2276397	ILMN_1726456		ILMN_1790228	ILMN_1784630
ILMN_1709085	ILMN_2375319	ILMN_1711608		ILMN_2331701	ILMN_1735779
ILMN_1750790	ILMN_1794594	ILMN_2125017		ILMN_3195401	ILMN_2130411
ILMN_1680937	ILMN_3235853	ILMN_3236289		ILMN_1761147	ILMN_1735930
ILMN_2231985	ILMN_1653504	ILMN_1691499		ILMN_3272074	ILMN_1768814
ILMN_3249645	ILMN_1705892	ILMN_2403006		ILMN_1718977	ILMN_1799134
ILMN_1670565	ILMN_1795118	ILMN_1724139		ILMN_1788802	ILMN_1797988
ILMN_1789074	ILMN_1678095	ILMN_1728106		ILMN_1812759	ILMN_2124920
ILMN_1660436	ILMN_1789913	ILMN_1801307		ILMN_2335813	ILMN_1658399
ILMN_1669927	ILMN_2152581	ILMN_1751464		ILMN_3276095	ILMN_1719093
ILMN_1801246	ILMN_1707005	ILMN_1788886		ILMN_1758906	ILMN_2213136
ILMN_1673352	ILMN_2367141	ILMN_1710427		ILMN_2176037	ILMN_1679185
ILMN_1805750	ILMN_1710746	ILMN_2224955		ILMN_1671237	ILMN_1723978
ILMN_2207291	ILMN_1683986	ILMN_1718792		ILMN_1656145	ILMN_1746148
ILMN_1667561	ILMN_1676470	ILMN_1666257		ILMN_1811171	ILMN_1812278
ILMN_1687390	ILMN_1677846			ILMN_1701947	ILMN_1731928
ILMN_2052511	ILMN_1731048			ILMN_1723274	ILMN_1756275
ILMN_2198878	ILMN_1654560			ILMN_1797728	ILMN_1675956
ILMN_3188500	ILMN_1741143			ILMN_1789074	ILMN_1669031
ILMN_1716234	ILMN_1723536			ILMN_1660436	ILMN_1663131
ILMN_1659913	ILMN_2306077			ILMN_1669927	ILMN_2147440
ILMN_1683927	ILMN_1688698			ILMN_1667561	ILMN_2147435
ILMN_2219246				ILMN_1687390	ILMN_2371433
ILMN_1751034				ILMN_1722209	ILMN_1761858
ILMN_1762080				ILMN_1786609	ILMN_1746025
ILMN_3251620				ILMN_1798204	ILMN_1689624
ILMN_1779857				ILMN_1746604	ILMN_2322986
ILMN_2137789				ILMN_1797861	ILMN_1655046
ILMN_1716093				ILMN_1849013	ILMN_1788955
ILMN_1665035				ILMN_1705737	ILMN_1738767
ILMN_1774088				ILMN_2198878	ILMN_2398274
ILMN_1770612				ILMN_3188500	ILMN_1698766
ILMN_1801632				ILMN_1716234	ILMN_1742026
ILMN_1801428				ILMN_1686989	ILMN_1669447

ILMN_1803788				ILMN_1793474	ILMN_2395981
ILMN_1730660				ILMN_1659913	ILMN_1677404
ILMN_1724062				ILMN_1664102	ILMN_1654586
ILMN_1713934				ILMN_1781099	ILMN_2276397
ILMN_1696749				ILMN_1785857	ILMN_2375319
ILMN_1737394				ILMN_1662128	ILMN_1794594
ILMN_1785527				ILMN_1683927	ILMN_1758941
ILMN_1754943				ILMN_1700432	ILMN_1796712
ILMN_2235851				ILMN_1683178	ILMN_2046730
ILMN_1707312				ILMN_1762080	ILMN_1684306
ILMN_1763560				ILMN_3251620	ILMN_1688780
ILMN_2408566				ILMN_2086077	ILMN_1713636
ILMN_2410145				ILMN_2397521	ILMN_3235853
ILMN_1661178				ILMN_2053415	ILMN_1653504
ILMN_2339955				ILMN_2355953	ILMN_1659158
ILMN_1782305				ILMN_1724062	ILMN_2258816
ILMN_1774586				ILMN_1713934	ILMN_2343618
ILMN_2382687				ILMN_1700413	ILMN_1755415
ILMN_1658163				ILMN_1680139	ILMN_1702787
ILMN_1807298				ILMN_1726164	ILMN_1788778
ILMN_1748591				ILMN_1758719	ILMN_1886719
ILMN_1887177				ILMN_2261076	ILMN_3229324
ILMN_1806725				ILMN_1743619	ILMN_3305938
ILMN_1775829				ILMN_2235851	ILMN_1702487
ILMN_3202109				ILMN_1707312	ILMN_1853711
ILMN_3251550				ILMN_2408566	ILMN_2387303
ILMN_1687978				ILMN_2410145	ILMN_1795118
ILMN_1659936				ILMN_1661178	ILMN_1653524
ILMN_1781073				ILMN_2339955	ILMN_1774806
ILMN_2363966				ILMN_1782305	ILMN_1678095
ILMN_1731589				ILMN_1774586	ILMN_1789913
ILMN_1696187				ILMN_2382687	ILMN_1694367
ILMN_2113049				ILMN_1658163	ILMN_1809478
ILMN_1741727				ILMN_1807298	ILMN_1815905
ILMN_1796677				ILMN_1718629	ILMN_1664956
ILMN_2316844				ILMN_2321578	ILMN_2152581
ILMN_1726913				ILMN_1784774	ILMN_3307252
ILMN_1708537				ILMN_2131381	ILMN_1708714
ILMN_2124064				ILMN_1791483	ILMN_1657796
ILMN_1766085				ILMN_1653125	ILMN_1750508
ILMN_1656011				ILMN_1691433	ILMN_1745593
ILMN_1733538				ILMN_1689057	ILMN_2294086
ILMN_1668559				ILMN_1760303	ILMN_1731048
ILMN_1811890				ILMN_2289433	ILMN_1654560
ILMN_2197365				ILMN_2398235	ILMN_1737705
ILMN_1802205				ILMN_2093343	ILMN_1651339
ILMN_1814494				ILMN_1653026	ILMN_1723536
ILMN_1654540				ILMN_1840334	ILMN_2306077
ILMN_1753251				ILMN_1679979	ILMN_2058251
ILMN_2317493				ILMN_1675858	ILMN_1782538
ILMN_1806407				ILMN_1734276	ILMN_1764573
ILMN_1717639				ILMN_2309156	ILMN_1688698
ILMN_3235647				ILMN_1803180	ILMN_1701875
ILMN_2098119				ILMN_1780898	ILMN_2371169
ILMN_1736834				ILMN_1696187	
ILMN_1651799				ILMN_2113049	
ILMN_1747197				ILMN_1741727	
ILMN_1726366				ILMN_2124064	
ILMN_2089329				ILMN_1766085	
ILMN_1728699				ILMN_1656011	
ILMN_1662618				ILMN_1733538	

ILMN_1756501				ILMN_1668559	
ILMN_2384496				ILMN_1811890	
ILMN_2048011				ILMN_2197365	
ILMN_1664859				ILMN_2381197	
ILMN_1802105				ILMN_1812327	
ILMN_2187206				ILMN_1657697	
ILMN_1785175				ILMN_1720889	
ILMN_1733318				ILMN_1689842	
ILMN_1702211				ILMN_1717639	
ILMN_1656761				ILMN_3235647	
ILMN_1754102				ILMN_2098119	
ILMN_2245292				ILMN_1736834	
ILMN_2318638				ILMN_1782938	
ILMN_2318643				ILMN_1769911	
ILMN_1765578				ILMN_2266948	
ILMN_1691499				ILMN_2317658	
ILMN_2403006				ILMN_1679041	
ILMN_1724139				ILMN_1726456	
ILMN_1728106				ILMN_1675429	
ILMN_1702691				ILMN_2203463	
ILMN_1764788				ILMN_1746029	
ILMN_1813379				ILMN_1791232	
ILMN_1801307				ILMN_1728699	
ILMN_1751464				ILMN_2041293	
ILMN_1686097				ILMN_1772241	
ILMN_1788886				ILMN_1711608	
ILMN_1684943				ILMN_1716224	
ILMN_1710427				ILMN_1663618	
ILMN_2224955				ILMN_2401978	
ILMN_1718792				ILMN_2410986	
ILMN_1666257				ILMN_1751871	
				ILMN_1731181	
				ILMN_1702211	
				ILMN_1656761	
				ILMN_1754102	
				ILMN_2245292	
				ILMN_2318638	
				ILMN_2318643	
				ILMN_2125017	
				ILMN_3236289	
				ILMN_1724139	
				ILMN_1702691	
				ILMN_2414325	
				ILMN_1749375	
				ILMN_1743100	
				ILMN_2349633	
				ILMN_1685005	
				ILMN_1764788	
				ILMN_1813379	
				ILMN_1748884	
				ILMN_1788886	
				ILMN_1684943	
				ILMN_2038775	
				ILMN_3211187	
				ILMN_3287687	
				ILMN_1710427	
				ILMN_2224955	
				ILMN_1718792	
				ILMN_1666257	
				ILMN_1762231	

Gene sets contain lists of genes, compiled in Illumina probe ID format, that are typically up- or downregulated in resident memory T cells (T_{RM}) from lung, skin and gut.
