# Supplementary material for: Transcriptional Analysis of T Cells Resident in Human Skin
Source: PLoS One. 2016 Jan 29;11(1):e0148351. doi: 10.1371/journal.pone.0148351 (PMC4732610; doi:10.1371/journal.pone.0148351)
Supplement: S3 Table — Significantly differentially expressed genes (DEGs) identified after pairwise comparison of microarray results with the RUVinv statistical method. Log2Fold-Change (log2FC) cutoff of 1.5 used. P<0.05 after multiple testing correction for all genes shown. Bold = differentially expressed genes shared between all 3 groups. (PDF) [file pone.0148351.s005.pdf]

S3 Table. Significantly differentially expressed genes between blood and skin T cells.

Blood vs Skin CD4		Blood vs Skin CD8		Blood vs Skin Treg	
Gene Symbol	log_2FC	Gene Symbol	log_2FC	Gene Symbol	log_2FC
HSPA7	-4.81	HSPA7	-4.52	ZSWIM4	-4.31
EGR1	-4.51	EGR1	-4.38	**RASD1**	-4.09
**NR4A1**	-4.17	**HSPH1**	-4.02	**HSPH1**	-4.04
**HSPH1**	-4.07	**NR4A1**	-3.99	SGK1	-3.80
MT2A	-3.86	**RASD1**	-3.83	**NR4A1**	-3.73
**PPP1R15A**	-3.40	TNF	-3.53	TNF	-3.69
BATF	-3.35	BAG3	-3.30	**FOS**	-3.42
**RASD1**	-3.28	**FOS**	-3.17	ANXA1	-3.28
**FOS**	-3.24	TUBA1C	-2.87	BAG3	-2.99
TSPYL2	-3.23	ZFAND5	-2.86	BHLHE40	-2.90
EGR2	-3.03	DUSP5	-2.69	CD40LG	-2.77
MT1X	-2.86	**PPP1R15A**	-2.57	**PPP1R15A**	-2.70
DNAJB1	-2.83	DNAJB1	-2.55	**GLA**	-2.60
UBE2S	-2.76	**ATF3**	-2.50	**NR4A2**	-2.51
PHLDA1	-2.74	SIK1	-2.40	ID2	-2.45
LDLR	-2.71	MT1X	-2.33	ATHL1	-2.39
SIK1	-2.61	**NR4A2**	-2.31	**ATF3**	-2.35
HSPB1	-2.55	DEDD2	-2.22	**PTGER4**	-2.23
SOCS1	-2.47	PIM2	-2.15	GPR183	-1.89
DEDD2	-2.45	GADD45B	-2.02	DB335527	-1.86
MT1E	-2.43	**GLA**	-1.98	PNP	-1.82
DDIT3	-2.41	CEBPB	-1.96	CD97	-1.82
LSMEM1	-2.41	**PTGER4**	-1.95	GTPBP2	-1.82
**CD69**	-2.32	CKS2	-1.93	**CD69**	-1.75
**NR4A2**	-2.29	GPR183	-1.89	NCOR2	-1.73
TTC13	-2.23	HSP90B1	-1.86	PRR5L	-1.69
EIF4A3	-2.22	PIP4K2A	-1.72	NFKB1	-1.63
**GLA**	-2.19	SAT1	-1.71	PAQR3	-1.61
CH25H	-2.18	CD40LG	-1.65	TARP	-1.55
DYNLL1	-2.16	UBB	-1.60	PGS1	1.51
CU680699	-2.12	DB335527	-1.59	SNORA7B	1.52
**PTGER4**	-2.09	SELK	-1.59	MED23	1.52
ZBTB48	-2.08	**CD69**	-1.52	FBXO18	1.57
NLRP3	-2.05	SRP9	1.52	SAP30L	1.58
APTX	-2.04	SLC38A1	1.59	ZFC3H1	1.60
CRIM1	-2.02	MPHOSPH8	1.61	**LEO1**	1.60
RAB11FIP5	-1.98	ATP5O	1.62	NDUFA4	1.63
HSPA8	-1.97	SEC16A	1.62	RALGAPA1	1.65
UBB	-1.84	SAP30L	1.64	UHMK1	1.67
ARHGAP9	-1.83	S1PR1	1.64	XPO4	1.67
CD97	-1.83	H3F3B	1.64	TACC1	1.68
SNORD52	-1.81	XPO4	1.64	SORL1	1.69

RSRP1	-1.81	PCMTD2	1.64	PPAP2A	1.74
SAT1	-1.80	SAMD3	1.65	IKZF2	1.75
**ATF3**	-1.77	C1GALT1	1.66	**ASB3**	1.75
NME3	-1.67	ZNF22	1.66	ARID4A	1.79
MT1G	-1.60	CCNY	1.68	CD47	1.82
SNORA22	-1.57	NKTR	1.68	MKRN1	1.83
SNORD68	-1.55	ZNF329	1.73	CLDND1	1.87
TRAF1	-1.54	CLDND1	1.75	ULK1	1.88
KLHL25	-1.53	**LEO1**	1.76	**TPP2**	1.89
H3F3C	-1.51	TSEN15	1.76	**TENM1**	1.91
SNAPC2	-1.50	**TPP2**	1.78	TIAL1	1.91
SETD8	1.50	ABLIM1	1.82	GPHN	1.94
CCDC12	1.51	**ASB3**	1.84	MDM2	1.94
CNEP1R1	1.52	**TSR2**	1.85	LEF1	1.95
GPRIN3	1.53	**KLF13**	1.87	**TSR2**	1.95
ATP5O	1.53	R3HDM2	1.87	R3HDM2	2.01
BX116997	1.54	**RNF10**	1.96	SNORD103B	2.01
UNK	1.54	GGA2	1.96	GTF3C6	2.02
TTC3	1.55	**FAM160B1**	1.97	NKTR	2.02
HEATR1	1.55	C18orf32	1.99	SELL	2.02
C6orf106	1.56	**SEC61A2**	2.08	PCMTD2	2.03
**ASB3**	1.58	RWDD1	2.14	**TSN**	2.05
TACC1	1.64	**RARRES3**	2.17	C18orf32	2.06
TCP11L2	1.65	**TSN**	2.20	**RNF10**	2.07
PLEKHA2	1.67	RNF126	2.20	**SEC61A2**	2.07
GPHN	1.68	SEC24C	2.21	ACSL5	2.10
CCNY	1.69	APPL2	2.24	**RARRES3**	2.11
**RARRES3**	1.74	**TENM1**	2.24	DKFZP586I1420	2.12
KANSL1L	1.76	ARIH2	2.27	RAB7A	2.13
**TSR2**	1.76	CASP8	2.37	LRIG1	2.14
MKRN1	1.77	**P2RY8**	2.37	CRLF3	2.16
SND1	1.81	NKG7	2.42	TXNDC12	2.17
C1GALT1	1.82	ASB1	2.45	LRRC8B	2.18
**TENM1**	1.84	**MASP2**	2.60	RBM39	2.19
UBE2G1	1.85	**ATP9B**	2.67	**KLF13**	2.20
FAM3C	1.85	NSG1	2.75	**P2RY8**	2.22
**LEO1**	1.86	PARGP1	2.80	MX1	2.24
SFT2D2	1.87	GMEB2	2.84	BECN1	2.31
S1PR1	1.96	CCR7	3.26	MIAT	2.45
PRPF4B	1.98	FURIN	3.96	ARIH2	2.46
AGFG1	1.98			ERP29	2.50
EIF4B	1.98			SEMA3G	2.51
**KLF13**	2.01			AGFG1	2.55
CD47	2.02			RWDD1	2.55
**TPP2**	2.03			**ATP9B**	2.89
**RNF10**	2.04			VAMP4	2.91
**SEC61A2**	2.05			COPB2	3.03
ALG5	2.07			PSMD7	3.03
CDK12	2.10			**MASP2**	3.20
TIAL1	2.11			**FAM160B1**	3.25

WBSCR22	2.11				
RASA3	2.16				
**P2RY8**	2.18				
SPTBN1	2.25				
**FAM160B1**	2.26				
**ATP9B**	2.27				
CNPPD1	2.34				
**TSN**	2.35				
ASB1	2.40				
APPL2	2.61				
CUX1	2.74				
EFR3A	2.82				
**MASP2**	2.95				

Significantly differentially expressed genes identified after pairwise comparison of microarray results with the RUVinv statistical method. Log_2Fold-Change (log_2FC) cutoff of 1.5 used. $P<0.05$ after multiple testing correction for all genes shown. Bold= differentially expressed genes shared between all 3 groups.
