# Supplementary material for: Transcriptional Analysis of T Cells Resident in Human Skin
Source: PLoS One. 2016 Jan 29;11(1):e0148351. doi: 10.1371/journal.pone.0148351 (PMC4732610; doi:10.1371/journal.pone.0148351)
Supplement: S4 Table — Significantly differentially expressed genes identified after pairwise comparison of microarray results with the RUVinv statistical method. Log2Fold-Change (log2FC) cutoff of 1.5 used. P<0.05 after multiple testing correction for all genes shown. Bold = common between blood and skin CD8 versus CD4 T cells. Bold italicized = common between blood and skin Treg versus CD4 T cells. (PDF) [file pone.0148351.s006.pdf]

S4 Table. Significantly differentially expressed genes between T cell lineages in blood and in skin.

Blood CD8 vs CD4		Skin CD8 vs CD4		Blood Treg vs CD4		Skin Treg vs CD4	
Gene	log_2FC	Gene	log_2FC	Gene	log_2FC	Gene	log_2FC
CD4	-1.85	ADAM15	-3.69	BHLHE40	-2.85	TRAPPC6A	-2.68
FAM125A	1.51	GSTM2	-2.63	ATHL1	-2.32	GSTM2	-2.62
C21orf2	1.52	KAT2B	-2.56	PDE4D	-2.26	ERP29	-2.53
CENPV	1.55	HIPK2	-2.48	FGF9	-2.25	EMP1	-2.40
PRF1	1.59	CASP8	-2.36	DENND5A	-2.21	RBM39	-2.38
**GNLY**	1.63	SNHG10	-2.22	ANXA1	-2.00	HIPK2	-2.29
CD8B	1.87	MB21D1	-2.04	AL359560	-1.96	SNHG10	-2.27
GZMH	1.89	NLRP3	-2.01	PELI2	-1.96	KAT2B	-2.13
**CST7**	1.98	CR611239	-2.00	IL4R	-1.83	NLRP3	-2.08
GZMA	1.99	FBXO11	-1.97	C1orf162	-1.82	ATL3	-2.06
MATK	2.16	HIST1H1E	-1.95	KIAA0531	-1.82	SLC20A1	-2.05
GZMK	2.69	TAGLN2	-1.93	TNF	-1.72	TAGLN2	-1.99
NKG7	3.20	NS4ATP2	-1.91	D4S234E	-1.71	CALM2	-1.95
**CD8A**	4.35	FBXW7	-1.89	PLAC8	-1.64	TCEB1	-1.95
		STAU1	-1.86	***RBMS1***	-1.57	EIF3E	-1.92
		RBM39	-1.83	CD40LG	-1.56	CR611239	-1.92
		ARIH2	-1.83	GNLY	-1.54	ARIH2	-1.90
		ATL3	-1.83	STAM	1.50	SPTAN1	-1.90
		CCR4	-1.76	DUSP10	1.53	***RBMS1***	-1.86
		OXNAD1	-1.75	IRF4	1.55	MB21D1	-1.84
		SNF8	-1.72	PCNA	1.56	MT1F	-1.84
		ESYT2	-1.71	HLA-DPA1	1.57	NS4ATP2	-1.83
		R3HDM2	-1.70	BCL2L11	1.62	PSMD7	-1.83
		TCEB1	-1.68	BTN2A2	1.65	NBPF14	-1.82
		KIAA1310	-1.68	FANK1	1.69	SNORD68	-1.81
		CAPN2	-1.66	OAS1	1.70	PPIG	-1.78
		PSMC1	-1.66	HLA-DMA	1.72	DDIT3	-1.77
		ATP1A1	-1.66	FLJ00326	1.73	G6PD	-1.76
		SNORD68	-1.59	SEMA3G	1.73	TAF7	-1.74
		PSMD7	-1.58	TMEM175	1.78	GLTSCR2	-1.72
		DDIT3	-1.55	BQ009175	1.84	CAPN2	-1.71
		LOC642852	-1.54	GBP5	1.84	RBM14	-1.71
		ADAM19	-1.54	TOX	1.90	ATP1A1	-1.71
		RBM14	-1.53	SGMS1	2.05	LPAR6	-1.71
		MCM7	-1.53	PMAIP1	2.19	LCP1	-1.69
		ARHGAP9	-1.52	TTN	2.32	SNF8	-1.69
		RORA	-1.50	IKZF2	2.44	R3HDM2	-1.66
		**CST7**	1.54	***CTLA4***	3.00	CSRNP1	-1.66
		ZFAND5	1.60	TRIB1	3.73	PSMC1	-1.65
		SNRPD2	1.70			LOC642852	-1.58
		LOC728855	1.74			KIAA1310	-1.57
		**GNLY**	1.78			ARHGAP9	-1.57

		**CD8A**	1.91			MRPL18	-1.57
						ADAM19	-1.56
						C12orf35	-1.55
						RPL32	-1.54
						PFDN5	-1.54
						RPL14	-1.51
						PEA15	1.55
						C21orf2	1.99
						CTLA4	2.73

Significantly differentially expressed genes identified after pairwise comparison of microarray results with the RUVinv statistical method. Log_2Fold-Change (log_2FC) cutoff of 1.5 used. $P<0.05$ after multiple testing correction for all genes shown. Bold= common between blood and skin CD8 versus CD4 T cells. Bold italicized= common between blood and skin Treg versus CD4 T cells.
