# Supplementary material for: Transcriptional Analysis of T Cells Resident in Human Skin
Source: PLoS One. 2016 Jan 29;11(1):e0148351. doi: 10.1371/journal.pone.0148351 (PMC4732610; doi:10.1371/journal.pone.0148351)
Supplement: S5 Table — Data obtained from PANTHER version 10.0 Overrepresentation Test (release 20150430) using PANTHER GO-Slim Biological Process annotation data set. P-values are adjusted for multiple testing with the Bonferroni method. (PDF) [file pone.0148351.s007.pdf]

S5 Table. Gene ontology (GO) analysis of differentially expressed genes upregulated in skin T cells compared to blood T cells.

GO-Slim Biological Process	# of genes involved	Expected representation	Fold Enrichment	Adjusted P-value
protein folding	7	0.56	> 5	0.000358
response to stress	12	2.37	> 5	0.000914
apoptotic process	9	1.97	4.56	0.0351
cell death	9	2.03	4.44	0.0424
death	9	2.04	4.41	0.0446
immune system process	15	5.01	2.99	0.0255

Data obtained from PANTHER version 10.0 Overrepresentation Test (release 20150430) using PANTHER GO-Slim Biological Process annotation data set. P-values are adjusted for multiple testing with the Bonferroni method.
