# Supplementary material for: Transcriptional Analysis of T Cells Resident in Human Skin
Source: PLoS One. 2016 Jan 29;11(1):e0148351. doi: 10.1371/journal.pone.0148351 (PMC4732610; doi:10.1371/journal.pone.0148351)
Supplement: S6 Table — Leading edge analysis was performed to determine which genes in the various skin T cell types contributed most to the enrichment score for the gene sets pertaining to skin resident memory T cells (TRM), i.e. gene sets containing the genes upregulated in skin TRM and downregulated in skin TRM. Treg = regulatory T cells. Bold = shared leading edge subset genes between the 3 groups. (PDF) [file pone.0148351.s008.pdf]

S6 Table. Results of leading edge analysis of Gene Set Enrichment Analysis.

CD8 leading edge subset		CD4 leading edge subset		Treg leading edge subset	
Upreg	Downreg	Upreg	Downreg	Upreg	Downreg
**AHR**	**ARAP2**	**AHR**	**ARAP2**	ADORA3	**ARAP2**
AMD1	**ARL2BP**	**ANXA1**	ARHGEF18	**AHR**	ARHGEF18
**ANXA1**	**BIN2**	ANXA2	**ARL2BP**	**ANXA1**	**ARL2BP**
CAPG	**CCL5**	CAPG	**BIN2**	**CD69**	**BIN2**
**CD69**	CD84	**CD69**	**CCL5**	CHN2	**CCL5**
CDKN1A	**DGKA**	CDKN1A	**DGKA**	CNTN1	CD84
CREM	**GIMAP4**	CREM	**GIMAP4**	CSF1	**DGKA**
CSRNP1	GIMAP6	CSRNP1	**GIMAP7**	**DUSP1**	**GIMAP4**
**DUSP1**	**GIMAP7**	DGAT1	**IFIT3**	**DUSP6**	GIMAP6
**DUSP6**	GMFG	**DUSP1**	**ITGB1**	**EGR1**	**GIMAP7**
**EGR1**	HEXB	**DUSP6**	**KLF3**	**EGR2**	GMFG
**EGR2**	**IFIT3**	**EGR1**	**KLHL24**	**ELL2**	**IFIT3**
**ELL2**	IL18RAP	**EGR2**	LEF1	FGL2	**ITGB1**
FAM174B	ITGAX	**ELL2**	LPIN1	**FOS**	**KLF3**
**FOS**	**ITGB1**	**FOS**	PLAC8	**FOSB**	**KLHL24**
**FOSB**	**KLF3**	**FOSB**	**PYHIN1**	**GADD45B**	LEF1
**GADD45B**	**KLHL24**	**GADD45B**	**RASA3**	**GEM**	LYST
GCH1	KLHL6	**GEM**	**RASGRP2**	**HSPA1A**	PRKCQ
**GEM**	KLRG1	GPR34	**S1PR1**	IFITM3	**PYHIN1**
GPR55	LPIN1	**HSPA1A**	**SH2D1A**	IFRD1	**RASA3**
**HSPA1A**	LYST	IFNG	**STK38**	**LMNA**	**RASGRP2**
IFNG	PLAC8	IFRD1	TCF7	**NFKBID**	**S1PR1**
KLF4	**PYHIN1**	IL13	**USP33**	**NR4A1**	**SH2D1A**
**LMNA**	**RASA3**	KLF4		**NR4A3**	SIDT1
NFIL3	**RASGRP2**	LGALS3		**PHLDA1**	**STK38**
**NFKBID**	**S1PR1**	**LMNA**		**PPP1R15A**	TLR1
**NR4A1**	**SH2D1A**	MYO1E		**RGS1**	**USP33**
NR4A2	**STK38**	NEURL3		**RGS2**	
**NR4A3**	TCF7	NFIL3		**SIK1**	
PERP	TLR1	**NFKBID**		**STYK1**	
**PHLDA1**	**USP33**	**NR4A1**		**TNF**	
**PPP1R15A**		NR4A2		TNFAIP3	
PTPN5		**NR4A3**		**TNFRSF9**	
QPCT		PERP		**TNFSF9**	
RASGEF1B		**PHLDA1**		VPS37B	
**RGS1**		**PPP1R15A**			
**RGS2**		PTPN5			
RHOB		PYGL			

**SIK1**		QPCT			
SPRY2		RASGEF1B			
SQSTM1		REL			
**STYK1**		**RGS1**			
TGIF1		**RGS2**			
**TNF**		RHOB			
**TNFRSF9**		**SIK1**			
**TNFSF9**		SQSTM1			
XCL1		**STYK1**			
		TGIF1			
		**TNF**			
		TNFAIP3			
		**TNFRSF9**			
		**TNFSF9**			
		XCL1			

Leading edge analysis was performed to determine which genes in the various skin T cell types contributed most to the enrichment score for the gene sets pertaining to skin resident memory T cells (T_{RM}), i.e. gene sets containing the genes upregulated in skin T_{RM} and downregulated in skin T_{RM}. Treg= regulatory T cells. Bold= shared leading edge subset genes between the 3 groups.
